# Artificial intelligence-based diagnostics of molar-incisor-hypomineralization (MIH) on intraoral photographs

**DOI:** 10.1007/s00784-022-04552-4

**Published:** 2022-05-24

**Authors:** Jule Schönewolf, Ole Meyer, Paula Engels, Anne Schlickenrieder, Reinhard Hickel, Volker Gruhn, Marc Hesenius, Jan Kühnisch

**Affiliations:** 1 Department of Conservative Dentistry and Periodontology, University Hospital, Ludwig-Maximilians University Munich, Goethestraße 70, 80336 Munich, Germany; 2 Institute for Software Engineering, University of Duisburg-Essen, Essen, Germany

**Keywords:** Chalky teeth, Automated image analysis, Convolutional neural networks, Deep learning, Transfer learning

## Abstract

**Objective:**

The aim of this study was to develop and validate a deep learning–based convolutional neural network (CNN) for the automated detection and categorization of teeth affected by molar-incisor-hypomineralization (MIH) on intraoral photographs.

**Materials and methods:**

The data set consisted of 3241 intraoral images (767 teeth with no MIH/no intervention, 76 with no MIH/atypical restoration, 742 with no MIH/sealant, 815 with demarcated opacity/no intervention, 158 with demarcated opacity/atypical restoration, 181 with demarcated opacity/sealant, 290 with enamel breakdown/no intervention, 169 with enamel breakdown/atypical restoration, and 43 with enamel breakdown/sealant). These images were divided into a training (*N* = 2596) and a test sample (*N* = 649). All images were evaluated by an expert group, and each diagnosis served as a reference standard for cyclic training and evaluation of the CNN (ResNeXt-101–32 × 8d). Statistical analysis included the calculation of contingency tables, areas under the receiver operating characteristic curve (AUCs) and saliency maps.

**Results:**

The developed CNN was able to categorize teeth with MIH correctly with an overall diagnostic accuracy of 95.2%. The overall SE and SP amounted to 78.6% and 97.3%, respectively, which indicate that the CNN performed better in healthy teeth compared to those with MIH. The AUC values ranging from 0.873 (enamel breakdown/sealant) to 0.994 (atypical restoration/no MIH).

**Conclusion:**

It was possible to categorize the majority of clinical photographs automatically by using a trained deep learning–based CNN with an acceptably high diagnostic accuracy.

**Clinical relevance:**

Artificial intelligence-based dental diagnostics may support dental diagnostics in the future regardless of the need to improve accuracy.

## Introduction

Visual examination is the method of choice for screening, monitoring, detecting, and diagnosing dental pathologies of teeth, and the corresponding diagnostic indices and methodological procedures have been described by the researchers [e.g., 1, 2]. However, the knowledge transfer from scientists to dental practitioners might sometimes be lacking, which is especially true for detecting and diagnosing individuals or teeth with molar-incisor-hypomineralization (MIH). Here, families notified diagnostic uncertainties by dental professionals which potentially results in conflicting positions, diverging recommendations and additional dental consultations [3, 4]. It might be beneficial to develop diagnostic methods to verify suspected dental hard tissue findings independently from the investigating dentist. In addition, this aim might be supported by the documented MIH prevalence rates. The mean global MIH prevalence was estimated recently at 13.1% by Schwendicke et al. [5]. In Germany, 28.7% of all 12-year-olds were found to have hypomineralizations [6, 7]. Both numbers indicate that a relevant proportion of adolescents is affected by this developmental disorder. Therefore, diagnosing and managing MIH is a frequent challenge in daily dental practice.

The aim of establishing independent diagnostic methods might become feasible by the availability of smart image analysis methods. Artificial intelligence (AI) currently offers the potential for the automated detection and evaluation of diagnostic information in medicine and dentistry [6–9]. The aim to digitalise medical and dental workflows must be understood as an emerging topic, and interest in this area has recently increased in dental research as well. Meanwhile, different workgroups have started to analyze all available types of dental radiographs [10–14] by using deep learning with convolutional neural networks (CNNs) for the detection of caries [15], apical pathologies [16], or periodontitis [17]. In contrast, only a few projects using AI-based algorithms for the automated identification of pathologies on intraoral clinical photographs have been reported [18–26]. When considering recently published reports and the latest software developments, it can be stated that, to the best of our knowledge, no application for the automated detection of MIH on intraoral photographs has been developed and/or evaluated thus far. Therefore, this diagnostic study aimed to train a CNN for MIH detection (test method); this CNN was then compared in its final stage to the expert evaluation (reference standard). The aim was to reach a diagnostic accuracy of at least 90% for the test method.

## Materials and methods

### Study design

**Fig. 1:**
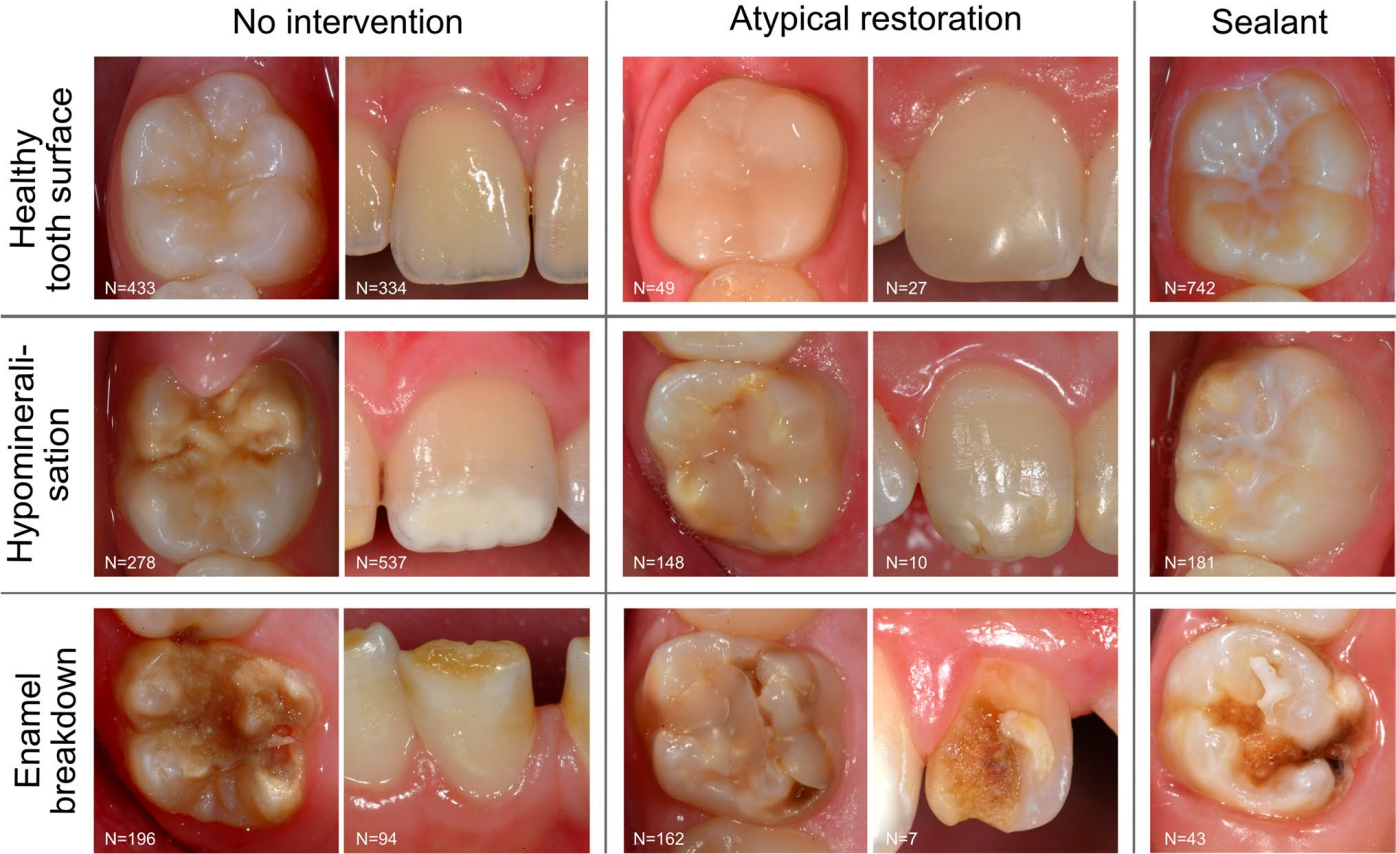
Overview of the chosen diagnostic categories based on the criteria provided by the European Academy of Paediatric Dentistry [3] and frequent intervention modalities

This diagnostic study used anonymized intraoral clinical photographs (Fig. 1) from clinical situations in which photographs were captured for educational purposes as well as from previously conducted clinical trials. The Ethics Committee of the Medical Faculty of the Ludwig-Maximilians University of Munich reviewed and approved the study concept (project number 020–798). This investigation was reported in accordance with the recommendations of the Standard for Reporting of Diagnostic Accuracy Studies (STARD) steering committee [27] and recently published recommendations for the reporting of AI studies in dentistry [28]. The pipeline of methods, mentioned below, was applied and described in previously published reports [19, 20].

### Intraoral photographs

Dental photographs were consistently taken with professional single-reflex cameras equipped with a 105-mm macro lens and a macro flash after tooth cleaning and drying [19, 20]. All images were stored (jpeg format, RGB colors, aspect ratio of 1:1) and selected for this study project. To ensure high data quality, duplicate or inadequate photographs, such as out-of-focus images, under- or overexposed pictures and photographs with saliva contamination, were excluded. Clinical photographs showing additional caries cavities and any other developmental disorders, e.g., amelogenesis or dentinogenesis imperfect or hypoplasia, were omitted. Caries-related restorations were also excluded to rule out potential evaluation bias. Finally, 3241 anonymized, high-quality clinical photographs from anterior and posterior permanent teeth with MIH (test group) and without any pathology/restoration (control group) were included in the study.

### Classification of teeth with MIH (reference standard)

Each photograph was classified with the goal of detecting and categorizing teeth with MIH in relation to the diagnostic classification system of the European Academy of Paediatric Dentistry [3] and possible dental interventions, such as restorations or fissure sealants. In detail, characteristics indicating the well-established MIH categories of demarcated opacities and enamel breakdowns are prevalent and can appear clinically in combination without any dental restoration, with an MIH-related—so called atypical—restoration or sealant (Fig. 1). Each image was precategorized by three graduated dentists (JS, PE, and AS) according to the given cross classification; afterwards, images were independently counterchecked by an experienced examiner (JK, > 20 years of clinical practice and scientific experience). In the case of divergent findings, each intraoral photograph was re-evaluated and discussed until consensus was reached. Every diagnostic decision—one per image—served as a reference standard for cyclic training and repeated evaluation of the deep learning-based CNN.

All the annotators were trained and calibrated before the study. During a 2-day theoretical and practical workshop guided by the principal investigator (JK), all annotators (JS, PE, and AS) were educated. Finally, 140 photographs were evaluated by all participating dentists to determine intra/interexaminer reproducibility for MIH classifications. Statistically, kappa values were computed for all coder pairs using Excel (Excel 2016, Microsoft, Redmond, WA, USA) and SPSS (SPSS Statistics 27, 2020, IBM corporation, Armonk, NY, USA). Intra/interexaminer reproducibility was calculated as 0.964/0.840–0.712 (JS), 0.982/0.747–0.727 (PE), 1.000/0.774–0.693 (AS), and 0.836/0.749–0.693 (JK), respectively. The documented kappa values indicated substantial to perfect agreement [29].

### Training of the deep learning-based CNN (test method)

In the following, the used pipeline of methods for developing the AI-based algorithm is described. Before training, the whole set of images (*N* = 3241) was divided into a training sample (*N* = 2596) and a test sample (*N* = 649); the CNN had no knowledge of the latter during training; it served as an independent test set only. The distribution of all images in relation to the diagnostic classification can be taken from Table 1.

**Table 1:** Description of the image set in relation to the diagnostic classification

Restoration status	MIH classification	Training sample	Test sample	Sum
No intervention	No MIH	627	140	767
	Demarcated opacity	659	156	815
	Enamel breakdown	232	58	290
Atypical restoration	No MIH	59	17	76
	Demarcated opacity	127	31	158
	Enamel breakdown	123	46	169
Sealant	No MIH	585	157	742
	Demarcated opacity	147	34	181
	Enamel breakdown	33	10	43
	Sum	2596	649	3241

To increase variability within the images, the underlying training set was augmented. For this purpose, the randomly selected images (batch size = 16) were multiplied by a factor of ~ 5, altered by different transformations (random center and margin cropping by up to 30% each; random deletion removing up to 30%; random affine transformation up to 180°; random perspective transformation up to a distortion of 0.5; and random changes in brightness, contrast, and saturation up to 10%) and resized (300 × 300 pixels). In addition, to compensate for under- and overexposure, all images were normalized [19, 20]. Torchvision (version 0.9.1, https://pytorch.org) in conjunction with the PyTorch library (version 1.8.1, https://pytorch.org) was used. ResNeXt-101–32 × 8d [30] was selected as the basis for the continuous adaptation of the CNN for MIH detection and categorization. The CNN was trained using backpropagation to determine the gradient for learning. Backpropagation was repeated iteratively for images and labels using the abovementioned batch size and parameters. Overfitting was prevented by two measures: selecting a low learning rate (0.0001) and performing dropout (at a rate of 0.5) on the final linear layers as a regularization technique. CNN training was repeated over 15 epochs with cross entropy loss as an error function and the application of the Adam optimizer (betas 0.9 and 0.999, epsilon 1e-8). With an open-source neural network employing pretrained weights (ResNeXt-101–32 × 8d pretrained on ImageNet, Stanford Vision and Learning Laboratory, Stanford University, Palo Alto, CA, USA), CNN training was accelerated. Existing learning results regarding the recognition of basic structures in the existing image set could thus be reused and skipped in the initial training. Training was performed on a university-based computer with the following specifications: RTX A6000 48 GB (Nvidia, Santa Clara, CA, USA); i9 10850 K 10 × 3.60 GHz (Intel Corp., Santa Clara, CA, USA) and 64 GB RAM [19, 20].

### Statistical analysis

The data were analyzed using Python (http://www.python.org, version 3.8). The overall diagnostic accuracy (ACC = (TNs + TPs)/(TNs + TPs + FNs + FPs)) was determined by calculating the number of true positives (TPs), false positives (FPs), true negatives (TNs), and false negatives (FNs). The sensitivity (SE), specificity (SP), positive and negative predictive values (PPVs and NPVs, respectively), and the area under the receiver operating characteristic (ROC) curve (AUC) were computed for the chosen MIH categorization [31]. Saliency maps were plotted to illustrate image areas that were used by the CNN to make individual decisions. The saliency maps were calculated by back propagating the CNN prediction and visualizing the gradient of the input of the resized images [19, 20, 32].

## Results

After the deep learning–based CNN was trained, the CNN was able to detect MIH and correlated interventions correctly in eight out of nine MIH categories with a diagnostic accuracy higher than 90% (Table 2). The overall diagnostic accuracy was determined at 95.2%. The SE and SP amounted to 78.6% and 97.3%, respectively. In detail, the accuracy values ranged from 91.5% (enamel breakdown/no intervention) to 99.1% (enamel breakdown/sealant). The lowest diagnostic accuracy of 88.4% was found for demarcated opacities with no intervention (Table 2). This was the only category—one out of nine—where the target accuracy of 90% was not reached (Table 2).

**Table 2:** Overview of the diagnostic performance of the developed convolutional neuronal network (CNN), where the independent test set (*n* = 649 images) was evaluated by the AI-based algorithm for the detection of MIH-related enamel disturbances and related interventions. The overall diagnostic accuracy (ACC, including the sensitivity (SE), the specificity (SP), the negative predictive value (NPV), the positive predictive value (PPV) and the area under the receiver operating characteristic curve (AUC)) was computed

Category		True positives (TPs)		True negatives (TNs)		False positives (FPs)		False negatives (FNs)		Diagnostic performance					
		N	%	N	%	N	%	N	%	ACC	SE	SP	PPV	NPV	AUC
No intervention	No MIH	128	19.7	485	74.7	24	3.7	12	1.9	94.5	91.4	95.3	84.2	97.6	0.985
	Demarcated opacity	116	17.9	458	70.6	35	5.4	40	6.1	88.4	74.4	92.9	76.8	92.0	0.922
	Enamel breakdown	37	5.7	557	85.9	34	5.2	21	3.2	91.5	63.8	94.3	52.1	96.4	0.901
Atypical restoration	No MIH	12	1.9	630	97.1	2	0.3	5	0.7	98.9	70.6	99.7	85.7	99.2	0.987
	Demarcated opacity	15	2.3	611	94.1	7	1.1	16	2.5	96.5	48.4	98.9	68.2	97.5	0.953
	Enamel breakdown	30	4.6	584	90.0	19	2.9	16	2.5	94.6	65.2	96.9	61.2	97.3	0.938
Sealant	No MIH	151	23.3	480	74.0	12	1.9	6	0.8	97.2	96.2	97.6	92.6	98.8	0.994
	Demarcated opacity	17	2.6	609	93.9	6	0.9	17	2.6	96.5	50.0	99.0	73.9	97.3	0.916
	Enamel breakdown	4	0.6	639	98.5	0	0	6	0.9	99.1	40.0	100.0	100.0	99.1	0.873
	∑	510	8.7	5053	86.5	139	2.4	139	2.4	95.2	78.6	97.3	78.6	97.3	n.c

*n.c.*, not calculable

When considering the diagnostic parameters of SE and SP in detail (Table 2), it is important to note that SP values were found to be consistently high, ranging from 92.9% (no intervention/demarcated opacity/) to 100.0% (sealant/enamel breakdown) in comparison to the SE. The latter ranged from 40.0% (enamel breakdown/sealant) to 96.2% (sealant/no MIH). The AUC values varied from 0.873 (enamel breakdown/sealant) to 0.994 (sealant/no MIH). With respect to the overall high AUC values, no ROC curves were plotted.

**Fig. 2:**
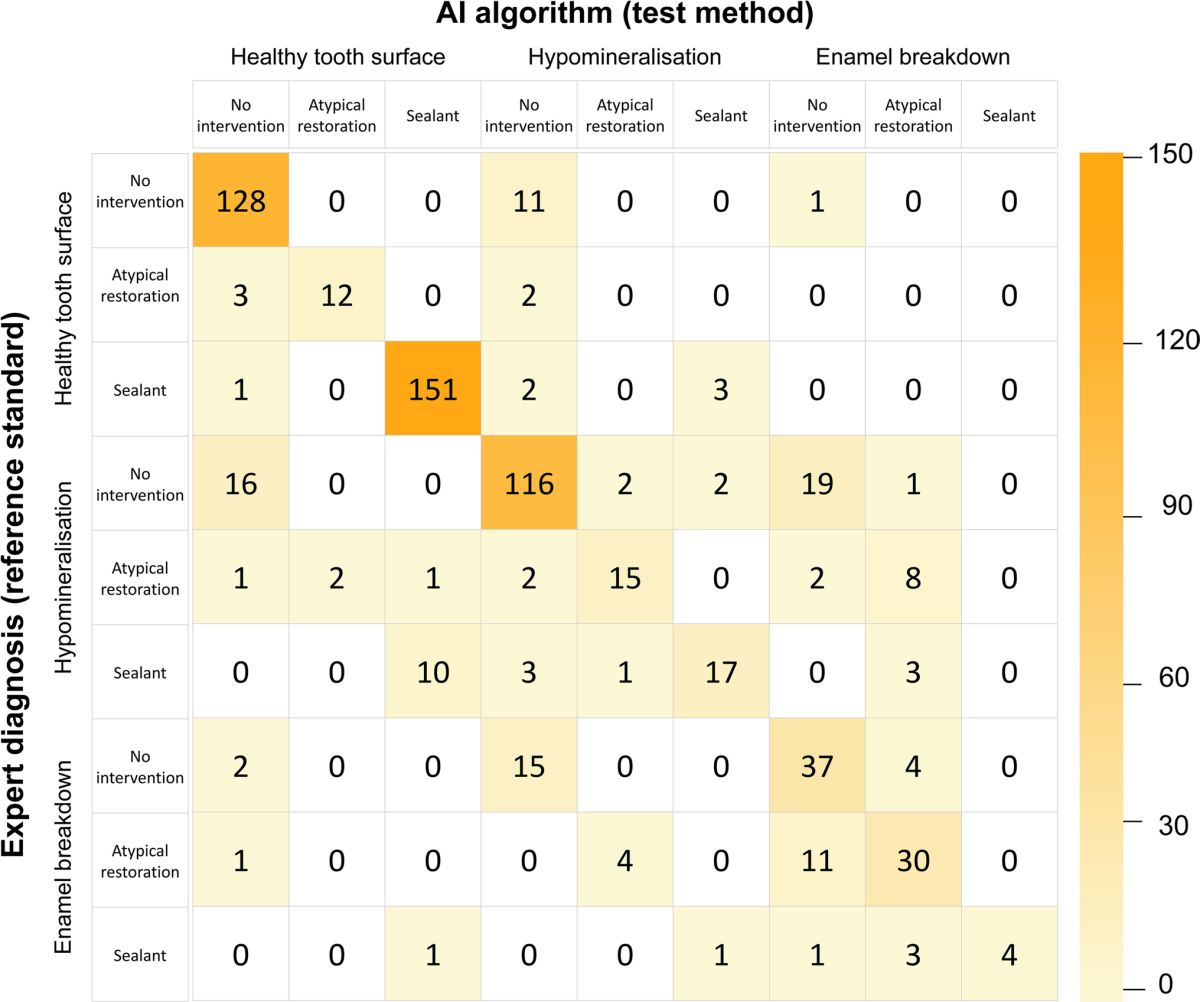
The confusion matrix shows the case distribution between the convolutional neuronal network (CNN, test method) and expert diagnosis for MIH assessment in the independent test set (*n* = 649 images)

**Fig. 3:**
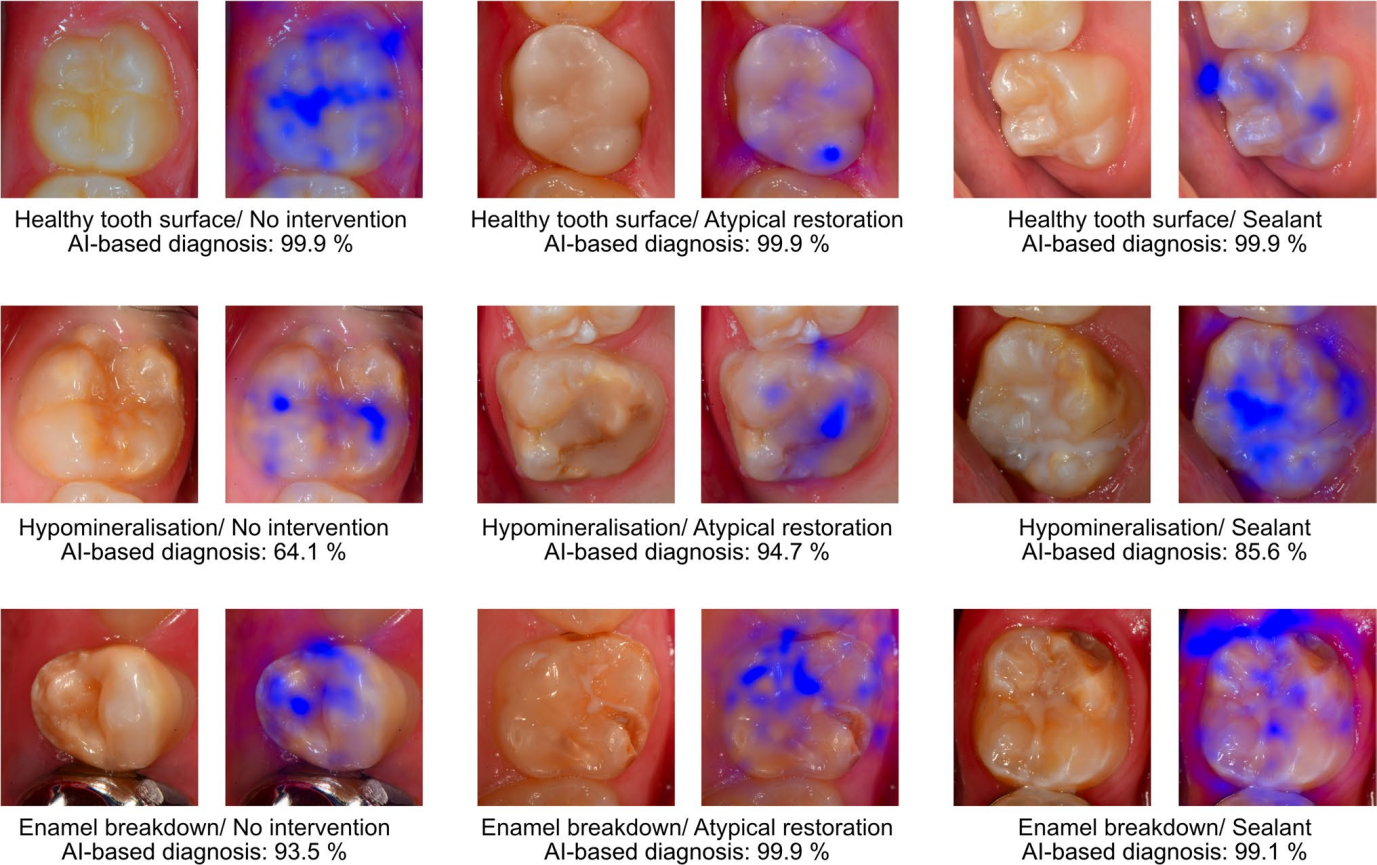
Example clinical images and the corresponding test results generated by the AI algorithms. Furthermore, the illustration includes saliency maps that depict those image areas (in blue) that the CNN used during the decision-making process

The confusion matrix (Fig. 2) illustrates the case distribution in the test set. Here, it also became obvious that the majority of diagnostic predictions by the AI-based algorithm (test method) were made in accordance with the expert decision in the test set. However, a distinct number of cases were not categorized correctly, especially if multiple characteristics were present on one photograph. In addition to the explorative data analysis, exemplary saliency maps (Fig. 3) are shown to illustrate areas on each intraoral photograph that the CNN used for decision-making.

## Discussion

The present diagnostic study demonstrated that an AI-based algorithm is able to detect MIH on intraoral photographs with a moderately high diagnostic accuracy (Table 2). With respect to the fact that accuracy > 90% was achieved in eight out of nine categories, the initially formulated hypothesis was accepted. When considering the documented accuracy and AUC values (Table 2), it could be further concluded that on the one hand, the overall diagnostic performance appears to be satisfactory but on the other hand, the partially low SE and high SP values indicate that the reported data need to be interpreted with caution. In detail, SP played a far more important role in this image sample probably because of the higher number of teeth without MIH. Therefore, the diagnostic accuracy is mainly driven by the SP rather than the SE and it could be argued that the AI-based algorithm is better in scoring sound teeth compared to MIH teeth. In this context, the complex clinical appearance of teeth with MIH, especially molars, needs to be highlighted. In addition to the fact that multiple findings can be present in teeth with MIH, this information will be further enhanced on intraoral photographs, which currently have a good resolution and can be thoroughly evaluated by the study team. Here, several demarcated opacities were found to have more or less extended enamel breakdowns that might be difficult to assess and for experienced clinicians to allocate to one of the given categories. While the experts allocated teeth with strictly small enamel breakdowns to this category, it can be taken from the saliency maps (Fig. 3) that the developed AI-based algorithm might have some difficulties in making such strict decisions as well. The same might be true for brighter demarcated opacities or small-sized atypical restorations, where the experts can provide precise assessments. To address and overcome this issue, appropriate pixelwise annotations must be recognized as a forward-looking methodological approach. But this also require a well-trained and well-calibrated annotator team as well as consistent quality controls to ensure correct diagnostic decisions. In the present study, the reproducibility was found to be in a good to excellent range. Additionally, the independent check of each diagnosis by an experienced dentists as well as consensus discussions and decisions completed the quality management.

Furthermore, the concept of transfer learning must be discussed. Contrary to earlier studies of our study group where only one diagnostic domain was included, e.g., caries [19] and sealant detection [20], the clinical complexity of teeth with MIH required the consideration of two domains with three diagnostic scores each and ultimately resulted in nine categories (Fig. 1). Pertinently, an imbalance of clinical cases is closely linked to the proposed cross-tabulated case categorization. Here, a few categories are underrepresented with respect to their rare presence in clinical practice. Therefore, the clinical variability of MIH characteristics as well as the low frequency of some categories probably impeded the training of the AI-based algorithm and may have lowered its overall diagnostic performance in comparison to the previously mentioned studies that used only a few diagnostic categories. To overcome this issue, the previously mentioned aspects of increasing the image data set and performing pixelwise annotations must be repeated. However, when considering the overall diagnostic performance of this initially developed AI-based algorithm for MIH categorization, the documented results (Table 2 and Fig. 2) should be interpreted as encouraging. Nevertheless, consistent future research is required.

Since no comparative studies or other AI-based methods are available for MIH diagnostics thus far, it is not possible to discuss this aspect specifically with respect to the current literature. However, it is feasible to consider results from other recently published diagnostic studies that used clinical photographs for the detection and categorization of dental findings. Here, a workgroup [21] published data for plaque detection on primary teeth, where an accuracy of 86.0% was reached. Noncavitated and cavitated caries lesions were detected with accuracies of 92.5% and 93.3%, respectively [19]. In other recently published diagnostic studies, white spot lesions were registered automatically with 81–84% accuracy [26] and caries lesions were classified and located with a mean AUC of 85.6% [33]. When also considering the available diagnostic performance data for various dental findings on different types of X-ray images [10–13, 15, 16, 18–20, 34, 35], it can be emphasized that the documented diagnostic accuracies in this trial are on the same order of magnitude compared to those of several other dental reports.

When summarizing the methodological strengths of this study project, it can be concluded that it was technically feasible to develop CNNs with substantial precision by using the described pipeline for software development. Therefore, it can be predicted that AI-based diagnostics will gain increasing attention in dentistry in the near future. However, further developments are needed before they can be used in a clinical setting [35, 36]. Moreover, it is crucial to assess the necessity of numerical extensive and qualitative image material to further improve the performance of the developed CNN for MIH categorization. Simultaneously, less frequent diagnostic categories should be included in appropriate numbers as well. Independently from this, it should further be noted that AI-based algorithms need to be also developed for rare developmental disorders, e.g., dentinogenesis or amelegenesis imperfecta. The chosen methodology primarily presents a simple approach to handle dental diagnoses and is typically linked with diagnostic accuracy values of approximately 90% (Table 2, Fig. 2). Aiming at increasing diagnostic performance up to 100%, the methodological requirements for consistent improvement of the data set and detailed image annotation by pixelwise labelling have been expressed. Another aim might be to perform CNN training on high-performance computers to reach a higher degree of neuronal connectivity. However, all these requirements will necessitate more time and personal and computing resources.

## Conclusion

It was possible in the present study to automatically categorize clinical photographs from teeth with MIH by using a trained deep learning-based CNN with an overall diagnostic accuracy of 95.2%. The higher NPV and SP values in comparison to PPV and SE indicate that the CNN performed better in healthy teeth compared to those with MIH. Future improvements are necessary to increase the diagnostic performance.

## Data Availability

The developed AI-based algorithm can be made available as a web application. In case of interest in our software solutions, please contact the author group or visit: https://dental-ai.de.

## References

[1] Lygidakis NA, Garot E, Somani C, Taylor GD, Rouas P, Wong FSL (2022). Best clinical practice guidance for clinicians dealing with children presenting with molar-incisor-hypomineralisation (MIH): an updated European Academy of Paediatric Dentistry policy document. Eur Arch Paediatr Dent.

[2] Lygidakis NA, Wong F, Jälevik B, Vierrou AM, Alaluusua S, Espelid I (2010). Best clinical practice guidance for clinicians dealing with children presenting with molar-incisor-hypomineralisation (MIH): an EAPD policy document. Eur Arch Paediatr Dent.

[3] Moreno T, Sanz JL, Melo M, Llena C (2021). Overtreatment in restorative dentistry: decision making by last-year dental students. Int J Environ Res Public Health.

[4] Gupta P, Gupta M, Koul N (2020). Overdiagnosis and overtreatment; how to deal with too much medicine. J Family Med Prim Care.

[5] Schwendicke F, Elhennawy K, Reda S, Bekes K, Manton DJ, Krois J (2018). Global burden of molar incisor hypominderalization. J Dent.

[6] Jordan AR, Micheelis W (2016). Fünfte Deutsche Mundgesundheitsstudie.

[7] Schwendicke F, Samek W, Krois J (2020). Artificial intelligence in dentistry: chances and challenges. J Dent Res.

[8] Khanagar SB, Al-Ehaideb A, Maganur PC, Vishwanathaiah S, Patil S, Baeshen HA, Sarode SC, Bhandi S (2021). Developments, application, and performance of artificial intelligence in dentistry - a systematic review. J Dent Sci.

[9] Grischke J, Johannsmeier L, Eich L, Griga L, Haddadin S (2020). Dentronics: towards robotics and artificial intelligence in dentistry. Dent Mater.

[10] Cantu AG, Gehrung S, Krois J, Chaurasia A, Rossi JG, Gaudin R, Elhennawy K, Schwendicke F (2020). Detecting caries lesions of different radiographic extension on bitewings using deep learning. J Dent.

[11] Krois J, Ekert T, Meinhold L, Golla T, Kharbot B, Wittemeier A, Dörfler C, Schwendicke F (2019). Deep learning for the radiographic detection of periodontal bone loss. Sci Rep.

[12] Kim JE, Nam NE, Shim JS, Jung YH, Cho BH, Hwang JJ (2020). Transfer learning via deep neural networks for implant fixture system classification using periapical radiographs. J Clin Med.

[13] Abdalla-Aslan R, Yeshua T, Kabla D, Leichter I, Nadler C (2020). An artificial intelligence system using machine-learning for automatic detection and classification of dental restorations in panoramic radiography. Oral Surg Oral Med Oral Pathol Oral Radiol.

[14] Schwendicke F, Elhennawy K, Paris S, Friebertshäuser P, Krois J (2020). Deep learning for caries lesion detection in near-infrared light transillumination images: a pilot study. J Dent.

[15] Lee JH, Kim DH, Jeong SN, Choi SH (2018). Detection and diagnosis of dental caries using a deep learning-based convolutional neural network algorithm. J Dent.

[16] Cha JY, Yoon HI, Yeo IS, Hun KH, Han JS (2021). Peri-implant bone loss measurement using a region-based convolutional neural network on dental periapical radiographs. J Clin Med.

[17] Lee JH, Kim DH, Jeong SN, Choi SH (2018). Diagnosis and prediction of periodontally compromised teeth using a deep learning-based convolutional neural network algorithm. J Periodontal Implant Sci.

[18] Li RZ, Zhu JX, Wang YY, Zhao SY, Peng CF, Sun Q, Hao AM, Li S, Wang Y, Xia B (2021). Development of a deep learning based prototype artificial intelligence system for the detection of dental caries in children. Zhonghua Kou Qiang Yi Xue Za Zhi.

[19] Kühnisch J, Meyer O, Hesenius M, Hickel R, Gruhn V (2022). Caries detection on intraoral images using artificial intelligence. J Dent Res.

[20] Schlickenrieder A, Meyer O, Schönewolf J, Engels P, Hickel R, Gruhn V, Hesenius M, Kühnisch J (2021). Automatized detection and categorization of fissure sealants from intraoral digital photographs using artificial intelligence. Diagnostics (Basel).

[21] You W, Hao A, Li S, Wang Y, Xia B (2020). Deep learning-based dental plaque detection on primary teeth: a comparison with clinical assessments. BMC Oral Health.

[22] Pauwels R (2021). A brief introduction to concepts and applications of artificial intelligence in dental imaging. Oral Radioln.

[23] You WZ, Hao AM, Li S, Zhang ZY, Li RZ, Sun RQ, Wang Y, Xia B (2021). Deep learning based dental plaque detection on permanent teeth and the influenced factors. Zhonghua Kou Qiang Yi Xue Za Zhi.

[24] Schwendicke F, Golla T, Dreher M, Krois J (2019). Convolutional neural network for dental image diagnostics: A scoping review. J Dent.

[25] Takahashi T, Nozaki K, Gonda T, Mameno T, Ikebe K (2021) Deep learning-based detection of dental prostheses and restorations. Sci Rep 11(1):1960. 10.1038/s41598-021-81202-x PMC7820223 33479303

[26] Askar H, Krois J, Rohrer C, Mertens S, Elhennawy K, Ottolenghi L, Mazur M, Paris S, Schwendicke F (2021). Detecting white spot lesions on dental photography using deep learning: a pilot study. J Dent.

[27] Bossuyt PM, Reitsma JB, Bruns DE, Gatsonis CA, Glasziou PP, Irwig L, Lijmer JG, Moher D, Rennie D, de Vet HC, Kressel HY, Rifai N, Golub RM, Altman DG, Hooft L, Korevaar DA, Cohen JF (2015). STARD 2015: an updated list of essential items for reporting diagnostic accuracy studies. BMJ.

[28] Schwendicke F, Singh T, Lee JH, Gaudin R, Chaurasia A, Wiegand T, Uribe S, Krois J (2021). Artificial intelligence in dental research: checklist for authors, reviewers, readers. J Dent.

[29] Landis JR, Koch GG (1977). The measurement of observer agreement for categorical data. Biometrics.

[30] Srivastava N, Hinton G, Krizhevsky A, Sutskever I, Salakhutdinov R (2014). Dropout: a simple way to prevent neural networks from overfitting. JMLR.

[31] Matthews DE, Farewell VT (2015). Using and understanding medical statistics.

[32] Simonyan K, Vedaldi A, Zisserman A (2021) Deep inside convolutional networks: visualising image classification models and saliency maps. In: In workshop at international conference on learning representations 2014. Accessed 31 January 2022. https://arxiv.org/pdf/1312.6034.pdf.

[33] Zhang X, Liang Y, Li W, Liu C, Gu D, Sun W (2022). Miao L (2022) Development and evaluation of deep learning for screening dental caries from oral photographs. Oral Dis.

[34] Bayraktar Y, Ayan E (2022). Diagnosis of interproximal caries lesions with deep convolutional neural network in digital bitewing radiographs. Clin Oral Investig.

[35] Lee S, Oh SI, Jo J, Kang S, Shin Y, Park JW (2021). Deep learning for early dental caries detection in bitewing radiographs. Sci Rep.

[36] Engels P, Meyer O, Schönewolf J, Schlickenrieder A, Hickel R, Hesenius M, Gruhn V, Kühnisch J (2022) Automated detection of posterior restorations in permanent teeth on intraoral photographs using artificial intelligence. J Dent 121:104124 10.1016/j.jdent.2022.104124 35395346

